# A case of immunoglobulin G4‐related aortic diseases

**DOI:** 10.1002/ccr3.1426

**Published:** 2018-02-13

**Authors:** Akira Sugaya, Yoshio Misawa, Shin‐ichi Ohki, Ippei Takazawa, Satoshi Uesugi

**Affiliations:** ^1^ Division of Cardiovascular Surgery Department of Surgery Jichi Medical University Shimotsuke Tochigi Japan

**Keywords:** Aortic aneurysm, aortic dissection, immunoglobulin G4‐related aortic disease

## Abstract

A 65‐year‐old man had histories of retroperitoneal fibrosis, membranous nephropathy, and acute coronary syndrome. Chest computed tomography showed an enlarged ascending aorta and type B aortic dissection, and he underwent ascending aorta and arch replacement. A pathological examination of the resected aorta showed immunoglobulin G4‐positive plasma cell infiltration.

## Introduction

Aortic dilatation including aortic dissection can be caused by different types of pathological conditions. Cystic medial necrosis, atherosclerosis, and other degenerative changes can lead to the aortic changes. Trauma and infection can also cause these aortic disorders. Immunoglobulin (Ig) G4‐related diseases are a new clinicopathological entity with unknown etiology. We report a case of aortic disorders caused by IgG4‐related aortopathy.

## Case History and Presentation

A 65‐year‐old man was admitted for treatment of an ascending aortic aneurysm and a chronic type B aortic dissection. He had experienced retroperitoneal fibrosis, membranous nephropathy, and acute coronary syndrome, 9, 7, and 4 years ago, respectively. A pathological examination did not indicate IgG4‐related diseases for retroperitoneal fibrosis and membranous nephropathy. Two‐year steroid therapy contributed to the relief of these conditions. IgG4 levels during the steroid therapy decreased to 19.8 mg/dL. Periarterial thickness and/or tumorous formation in a coronary angiogram, which indicated IgG4‐related angiopathy, were not clear. The patient underwent percutaneous coronary intervention. When he developed type B aortic dissection 3 years ago, his serum IgG4 level was 190 mg/dL.

**Figure 1:**
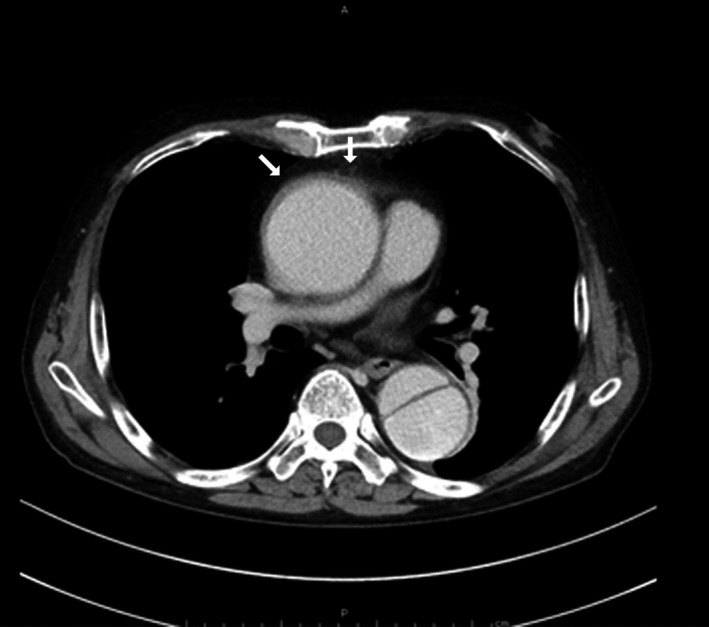
Contrast‐enhanced chest computed tomography. A dilatation of the ascending aorta and a dissection of the descending aorta are observed. The soft tissue around the ascending aorta is thickened showing irregular surfaces (arrows).

A physical examination on admission showed no remarkable findings. A blood test showed a slightly elevated creatinine level of 1.46 mg/dL with an estimated glomerular filtration rate of 39 mL/min/1.73 m^2^. Liver function was within the normal limit without anemia. An ultrasonic cardiogram showed no valvular diseases associated with dilatation of the ascending aorta. Chest computed tomography showed an enlarged ascending aorta of 62 mm in diameter and type B aortic dissection between the distal aortic arch and the bilateral common iliac arteries (Fig. 1). Thickened periaortic changes were detected. The celiac trunk, superior mesenteric artery, and right renal artery originated from the true lumen, and the left renal artery originated from the pseudolumen.

**Figure 2:**
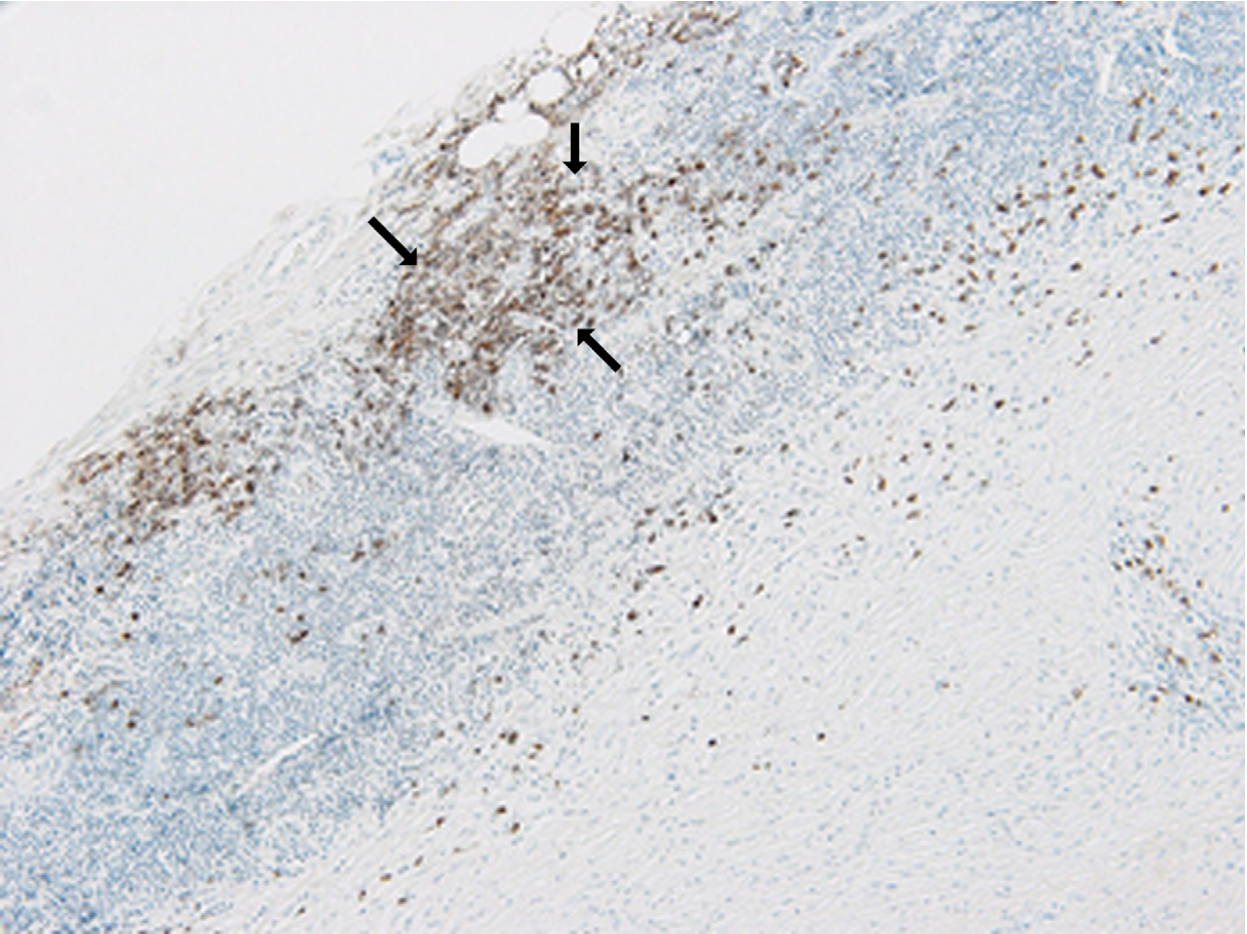
Pathological findings of the ascending aorta. Immunohistochemical staining for IgG 4. The adventitia shows severe inflammatory changes with IgG4‐positive plasma cell infiltration (arrows) associated with dense fibrous tissue. The IgG4‐positive cells occupy more than 50% among the total plasma cells.

The patient underwent ascending aorta, and arch replacement associated with open stent implantation in the descending aorta through a standard full sternotomy. The stent graft was positioned at 3 cm distal from the entry of the descending aorta. Operative findings included periaortic thickness and edematous changes. The intima was thickened without atheromatous changes. A pathological examination showed cystic medial necrosis without atherosclerotic changes (Fig. 2). The adventitia showed severe inflammatory changes with IgG4‐positive plasma cell infiltration. The IgG4‐positive cells occupied more than 50% among the total plasma cells. The postoperative serum IgG4 level was 48.5 mg/dL. The pathological findings and high serum IgG4 levels suggested that IgG4‐related aortopathy had caused the aortic dissection and aortic dilatation.

The patient's postoperative course was uneventful. He was doing well almost 1 year after the operation without newly developed organ dysfunction, and his serum IgG4 level was within the normal limit without steroid therapy.

## Discussion

IgG4‐related diseases are newly recognized systemic disorders, and they can affect a variety of different organs. IgG4‐related inflammatory changes can also cause different morphological changes in the aorta 1, 2, 3, 4, 5, 6, 7, 8.

Lindsay and colleagues reported a case of periaortitis 1. An abdominal CT scan of the case revealed periaortic soft tissue surrounding the abdominal aorta. The soft tissue extended to include the proximal half of each common iliac artery. No calcification or aneurysmal changes were observed. A biopsy of the periaortic mass led to the conclusion that more than 50% of the plasma cells were positive for IgG4.

Periaortitis can progress to aortic rupture. Ikeda and Kasashima reported a case of IgG4‐related periaortitis that was complicated by aortic rupture 2, 3. Patients with IgG4‐related aortitis are older than those with Takayasu's arteritis or Behcet's aortitis and are more likely to be male than female compared with Takayasu's arteritis 4.

Aneurysmal changes, as shown in our case, can also occur. Nuñez Fernández and colleagues described a case of aneurysmal dilatation of the ascending aorta 5. Diagnosis of IgG4‐related aneurysmal changes is made by immunohistochemistry, with greater than 50% of IgG4‐positive cells and an IgG4/IgG cell ratio greater than 0.4. Colombier and colleagues reported a similar case to our case with an asymptomatic ascending aortic aneurysm and thickening of the aortic wall 7. IgG4‐related cardiovascular diseases affect the coronary arteries, heart valves, myocardium, pericardium, aorta, and peripheral vasculature. A case of abdominal aortic aneurysm associated with a coronary artery aneurysm and periarteritis has also been reported 8. Additionally, periaortic inflammatory change might precede aortoduodenal fistula formation 9.

In our case, pathological findings of retroperitoneal fibrosis and membranous nephropathy or coronary angiographic findings of acute coronary syndrome were not consistent with IgG4‐related diseases. Additionally, the resected specimen for pathological examination was obtained from the ascending aorta and aortic arch alone. However, we concluded that the aortic dissection and aortic aneurysm were caused by IgG4‐related aortopathy.

## Conclusions

We present a case of aortic dissection and aortic aneurysm in a 65‐year‐old man. He had experienced retroperitoneal fibrosis, membranous nephropathy, and acute coronary syndrome. He underwent ascending aorta and arch replacement associated with open stent implantation in the descending aorta. Pathological examination of the adventitia showed severe inflammatory changes with IgG4‐positive plasma cell infiltration. We conclude that the aortic dissection and aortic aneurysm were caused by IgG4‐related aortopathy in our patient.

## Conflict of Interest

None declared.

## Authorship

YM: designed study conception. AS and SO: helped in data collection. AS: performed analysis. SO, IT, and SU: helped in investigation. AS and YM: helped in writing.
